# Opening closed doors: using machine learning to explore factors associated with marital sexual violence in a cross-sectional study from India

**DOI:** 10.1136/bmjopen-2021-053603

**Published:** 2021-12-30

**Authors:** Lotus McDougal, Nabamallika Dehingia, Nandita Bhan, Abhishek Singh, Julian McAuley, Anita Raj

**Affiliations:** 1 Center on Gender Equity and Health, University of California San Diego, La Jolla, California, USA; 2 Public Health & Mortality Studies, International Institute for Population Sciences, Mumbai, Maharashtra, India; 3 Department of Computer Science, University of California San Diego, La Jolla, California, USA; 4 Department of Education Studies, University of California San Diego, La Jolla, California, USA

**Keywords:** public health, social medicine

## Abstract

**Objectives:**

Sexual violence against women is pervasive in India. Most of this violence is experienced in the context of marriage, and rates of marital sexual violence (MSV) have been relatively stagnant over the past decade. This paper machine learning algorithms paired with qualitative thematic analysis to identify new and potentially modifiable factors influencing MSV in India.

**Design, setting and participants:**

This cross-sectional analysis of secondary data used data from in-person interviews with ever-married women aged 15–49 who responded to gender-based violence questions in the nationally representative 2015–2016 National Family Health Survey (N=66 013), collected between 20 January 2015 and 4 December 2016. Analyses included iterative thematic analysis (L-1 regularised regression followed by iterative qualitative thematic coding of L-2 regularised regression results) and neural network modelling.

**Outcome measure:**

Participants reported their experiences of sexual violence perpetrated by their current (or most recent) husband in the previous 12 months. These responses were aggregated into any vs no recent MSV.

**Results:**

Nearly 7% of women experienced MSV in the past 12 months. Major themes associated with MSV through iterative thematic analysis included experiences of/exposure to violence, sexual behaviour, decision making and freedom of movement, sociodemographics, access to media, health knowledge, health system interaction, partner control, economic agency, reproductive and maternal history, and health status. A neural network model identified variables that largely corresponded to these themes.

**Conclusions:**

This analysis identified several themes that may be promising avenues to identify and support women experiencing MSV, and to mitigate these traumatic experiences. In particular, amplifying screening activities at health encounters, especially among women who appear to have compromised health or restricted agency, may enable a greater number of women access to essential physical and emotional support services, and merits further consideration.

**Strengths and limitations of this study**

- The sample size and design of these data enable nationally representative exploration of themes associated with marital sexual violence (MSV) among more than 66 000 Indian women, each of whom is represented by more than 5500 variables.
- This analysis applies an innovative, blended use of machine learning and qualitative research methods in an iterative thematic analysis approach previously validated on these same data.
- This analysis could be further strengthened by the addition of more detailed information on MSV, including the place and specific circumstances of perpetration.
- The cross-sectional, observational design precludes causal inference.

## Introduction

Sexual violence is a prevalent and pervasive violation of health and human rights, and has been the focus of global mitigation campaigns and policies.^1–3^ This issue is of great importance in India, where highly publicised cases of sexual violence against women have led to public awareness and landmark changes in laws and reporting of violence.^4 5^ Despite this, 6% of Indian women have experienced sexual violence in their lifetimes.^6^ This is higher than the global average of 4.2%, and the vast majority of this abuse is reported as perpetrated by intimate partners, primarily husbands.^6 7^ At current population estimates, these amount to an estimated 13.6 million Indian women between the ages of 15 and 49 who report having experienced sexual abuse by their spouses in the past year.^6 8^

Despite initiatives designed to address this substantive burden, reductions in marital sexual violence (MSV) in India have been slow, declining only 2% between 2005–2006 and 2015–2016.^6 9^ Several factors have contributed to this stagnation, including challenges related to legal frameworks around marital rape in India.^10 11^ Stigmatisation, fear of retribution, and inadequate or inappropriate medical and psychological response services contribute to extremely low levels of disclosure (<10% of victims of sexual violence told anyone about their experiences), limiting the support accessed by victims.^6 12 13^ Underlying and sustaining this entire process are fundamentally imbalanced gender norms and issues of power in intimate relationships that devalue women and girls.^14–16^

MSV in India is thus an injustice for which there are limited legal protections at present, patchy structural response and support services, and pervasive social barriers around disclosure and redressal.^17 18^ To date, the focus of research on MSV has predominantly been on understanding its prevalence, sociodemographic correlates and its relationship with earlier marriage of girls, providing insights into the social and spatial distribution of the issue in India.^4 12 19–22^ A major gap in our understanding at present is in examining the role of women’s agency and key determinants within the couple’s relationship that might explain this issue. Understanding these key factors are important not just from the prevalence perspective, but also to identify the pathways and intervention points to expand social and health services for women affected by MSV and where existing mitigation and support services may be particularly effective.

The objective of this study was to use machine learning to explore an expansive array of social, demographic and health factors available in a large national survey to determine which of these are associated with MSV, with the goal of identifying previously unknown and potentially modifiable factors related to this outcome. Such identification can offer new insights into potential mechanisms for change to reduce or eliminate MSV in India. We use a novel approach that blends machine learning modelling with qualitative research techniques, to identify issues and insights that are meaningful to the issue of MSV with content and context considerations.

## Methods

Data were drawn from the 2015–2016 India National Family Health Survey (NFHS-4), a nationally representative survey collecting information on a variety of topics related to health and sociodemographics from women aged 15–49.^6^ NFHS-4 data are publicly available from the Demographic and Health Survey (DHS) programme.^23^ NFHS-4 was implemented as stratified, two-stage survey design with sex-matched survey enumerators interviewing respondents in 601 509 households across all states and union territories in India. Women were eligible to be interviewed if they were between ages 15–49, and stayed in the selected household the night before their interview (n=6 99 686, response rate=96.7%). In a subset of 15% of selected households, one eligible women was randomly selected to answer questions on gender-based violence, and was interviewed in private in accordance with WHO guidelines^24^ (n=79 729, response rate=95.6%). The 4.4% of women who were selected for gender-based violence interviews, but not interviewed, were nearly universally attributed to the lack of a private interview location. As the outcome of interest is marital violence, our analytic sample comprised ever-married women aged 15–49 who responded to gender-based violence questions (N=66 013).

### Measures

Our outcome of interest was experience of recent MSV, categorised as ‘any’ versus ‘none’. This was based on women’s responses regarding whether their current (or most recent) husband had perpetrated any of the following acts in the previous 12 months: being physically forced into unwanted sex, being physically forced to perform sexual acts the woman did not want to or being forced into other unwanted sexual acts.

All other survey response variables from the NFHS-4 dataset were included in the statistical models as independent variables, or features. To prepare the dataset for machine learning algorithms, all categorical variables with three or more levels were one-hot encoded (ie, transformed into multiple binary variables^25^), and all continuous variables were normalised. Variables that represented the same information in different ways (eg, both categorical and continuous representations of age) were identified and removed so that each measure was included only once in the analysis. In total, each woman was represented as a set of 5549 features.

### Statistical analysis

Our study followed a previously tested methodology for identification of potential correlates of an outcome using a dataset with a large number of independent variables or features. The original study used NFHS-4 data to test and validate this approach using three different types of machine learning models: L-1 regularised regression or lasso, L-2 regularised regression, or ridge, and neural network.^26^

We used the same three types of regression-based supervised learning models to identify features included in the NFHS-4 dataset which are associated with MSV. Supervised learning models are a family of machine learning algorithms used when there is a specific outcome of interest, that is, a dependent variable,^27^ and involve dividing the data into three sets for training, validation and testing. The training set is used to build the model and optimise coefficient values, the validation set is used to estimate tuning parameters (including regularisation), and the test set is used to evaluate the built model and estimate accuracy and error rates. We randomly split the NFHS-4 dataset into training set (80%) and test set (20%). For validation, rather than segregating an additional portion of the original dataset, fivefold cross-validation was used on the training sets for each of the machine learning models.^28^

#### Machine learning models

We used two types of regularised machine learning models in our study: L-1 regularised, and L-2 regularised models. Regularisation is a form of regression that imposes a penalty on the size of logistic regression coefficients, shrinking them towards zero to avoid overfitting. Unlike traditional regression models used in epidemiology or public health research, which can often be vulnerable to multi-collinearity and overfitting, regularised models can offer accurate and relevant results when dealing with a large number of features. L-1 regularised logistic regression, or lasso, is often used when building models with datasets that have a large number of features, as it can serve as a data reduction model. Lasso logistic regression can be represented as follows:

$$l_{\theta }\left(y|X\right)=\sum_{i}-log\left(1+e^{-X_{i}\theta }\right)+\sum_{y_{i}=0}-X_{i}\cdot \theta -\lambda \|\theta {\|}_{1}$$

Where *l* is the likelihood function, *y* is the outcome, *X* is the vector matrix of all independent variables, *θ* is the vector matrix of all coefficients of the independent variables, *λ* is the regularisation parameter and $\lambda \|\theta {\|}_{1}$ is thus the penalty term. Lasso sets a constraint parameter on coefficient size in a regression model, which shrinks the coefficients toward zero.^29^ Because the penalty parameter is the sum of the absolute value of the coefficients, those least relevant to the outcome in terms of their strength of association, are set equal to zero and drop out of the analysis. Lasso is therefore used to reduce dimensionality in large datasets, as well as to lessen multicollinearity in the final models.

The L-2 regularised logistic regression, or ridge, model also uses regularisation to estimate outcome probabilities. Ridge follows a similar log-likelihood equation as lasso, with the exception of the penalty term, which is instead represented by $\lambda \|\theta {\|}_{2}^{2}$ . Because ridge models include this squared coefficient term, no coefficient values can reduce to zero. Thus, the key difference between lasso and ridge regression model is that lasso shrinks the less important features’ coefficient values to zero, while ridge does not. Lasso is an apt choice when we want to identify irrelevant or less important features in terms of their lack of association with an outcome of interest.

Neural networks are more complex models, which, unlike the logistic regression models, do not assume a strictly linear relationship between the features and the outcome.^30^ The artificial neurons in the machine learning neural network models are essentially the inputs, or the independent variables, multiplied by weights, and passed through a certain activation function.^31^ These activated outputs are then passed on to the next layer of artificial neurons, known as hidden layers. The process continues until the final layer is the output, or the dependent variable. Using a learning algorithm, the neural net modifies weights for each layer of the neural network model. For our study, we used a neural network model with four hidden layers.

While there are multiple types of machine learning models, our choice of regularised and neural network models was guided by discussions among gender research and computer science experts, as well as previous testing in these data.^26^ The selected three algorithms are appropriate for our research question, and are routinely used and robust methods that have been commonly used for classification tasks.

#### Analytic approach

The three machine learning models were implemented using two strategies: (a) L-1 regularised regression model followed by L-2 regression model, with iterative qualitative categorisation and (b) L-1 regularised regression followed by neural network. Individual correlates of MSV were identified separately from the two strategies. All models were evaluated using the area under receiver operating characteristic curve (AUC), and balanced error rate (BER). These evaluation metrics take into account both sensitivity and specificity, and are apt for imbalanced datasets like ours, where the prevalence of the outcome is low.

**Figure 1:**
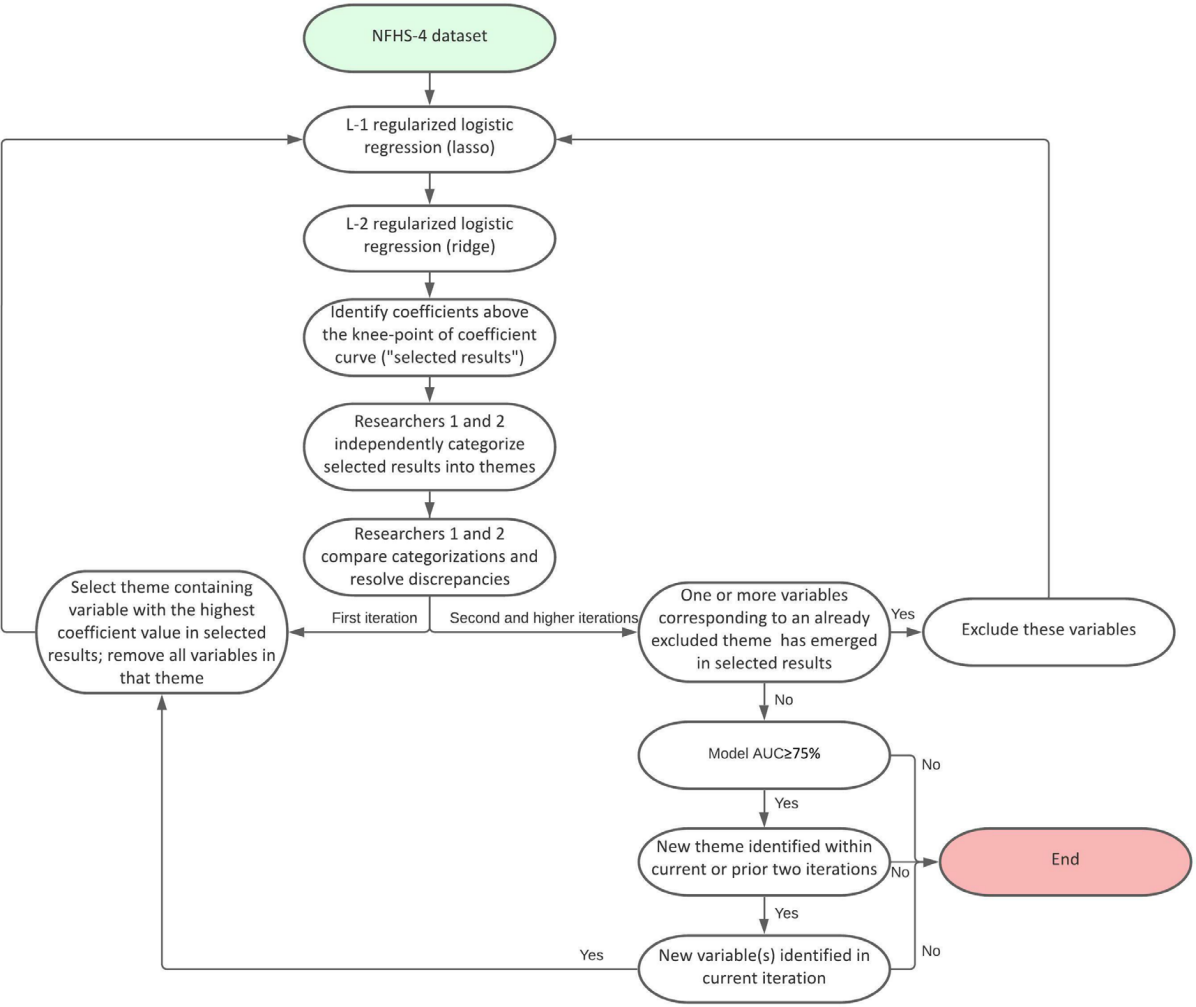
Flowchart for iterative thematic analysis of variables associated with marital sexual violence. NFHS-4, National Family Health Survey; AUC, area under receiver operating characteristic curve.

Our objective was to identify multiple underlying constructs associated with MSV, with no predefined constraints on variables of interest. We thus adopt an iterative thematic analysis approach that blends hypothesis-generating methodologies of qualitative research with machine learning models in order to identify key concepts or domains associated with an outcome, as opposed to individual variables (figure 1).^26^ This approach qualitatively reviews machine learning model results to identify coherent and relevant themes within the resultant set of independent variables. For example, variables measuring household wealth and religion were grouped and coded as a socio-demographic theme.

As outlined in figure 1, iterative thematic analysis began with a lasso, or an L-1 regularised model. Using lasso prior to ridge allowed for the inclusion of a parsimonious ridge model, excluding variables that were unrelated to our outcome of interest, MSV as defined by coefficient values of zero. The ridge logistic regression results were ranked by coefficient values, given that most of our features were dichotomous variables; the higher the coefficient value, the higher its importance in terms of its strength of association with MSV. We selected the top-most variables which had coefficient values higher than the knee point of the coefficient curve, using the mathematical definition of curvature for a continuous variable as the basis of the knee-point for our analysis (online supplemental figure 1).^32^

10.1136/bmjopen-2021-053603.supp1 Supplementary data

These variables above the knee point of the coefficient curve were then independently coded into themes by two researchers. Two or more variables were considered a theme when they were topically related and the number of variables within the theme was at least 5% of identified variables. The theme with variables that had highest variance, or coefficient values then excluded, and the cycle of lasso, ridge and thematic coding was repeated. In cases where a variable was related to multiple themes, it was excluded from the model only when all the themes it corresponded to were excluded. When new variables emerged that would have been coded into a previously excluded theme, they were excluded. Iterative thematic analysis was continued until (a) model AUC dropped below 75%, (b) no new themes were identified for three consecutive rounds or (c) no new variables were identified in the most recent round.

The coding by the two researchers were matched for any disagreements and a final set of themes were decided. We found a percent agreement rate of >95% between the two researchers, in each round of individual categorisation.

Our second strategy to identify correlates of MSV used neural network model, accounting for potential non-linear relationships among the independent variables. To ensure the use of a parsimonious model, we first implemented lasso, and dropped the variables with a resultant coefficient value of zero. The variables with a non-zero coefficient from lasso were included as inputs to the neural network model. The results from the neural network model were ranked in terms of ‘variable importance’, which was defined as the value of coefficient weights for the first hidden layer. We selected the variables with coefficient values higher than the knee-point of the coefficient curve as our key correlates of MSV.

All analyses were conducted using Python with necessary libraries (pandas, scipy, keras, numpy, sklearn) and women’s individual domestic violence weights. The use of these domestic violence weights accounts for the fact that only one woman in a subset of households was eligible to respond to the gender-based violence questions, and thus allows analyses to be nationally-representative.

### Patient and public involvement

This study involved secondary analysis of publicly available, de-identified survey data; no patients were directly involved in this study. As secondary data users, it was not possible to contact survey respondents to involve them in the design, conduct, reporting, or dissemination plans of our research.

## Results

### Sample characteristics

Of the 66 013 eligible, ever-married women aged 15–49 in our sample, nearly 6% had experienced MSV in the past 12 months (table 1). Interviewed women were an average of 33 years old, and two-thirds (66%) lived in rural areas. Post-secondary education was rare (10% in the overall sample, 5% among women who experienced recent MSV). Among women who recently experienced MSV, there was a higher proportion of SC/STs (37% vs 29% in the overall sample) and of low household wealth (29% in the poorest household wealth quintile vs 18% in the overall sample).

**Table 1:** Sociodemographic characteristics of analytic sample of women aged 15–49 in India, 2015–2016

	All women (n=66 013)	Recent marital sexual violence (n=4372)
Characteristics	Wtd. %/mean	Wtd. %/mean
Total	100%	5.7%*
Age	33.1	32.9
Literacy	61.4%	51.8%
Education		
None	33.8%	43.2%
Primary	14.4%	17.1%
Secondary	41.9%	35.1%
Higher	9.8%	4.6%
Household wealth quintile		
Poorest	18.3%	29.1%
Poorer	19.8%	22.6%
Middle	20.6%	21.4%
Richer	20.9%	15.9%
Richest	20.3%	10.9%
Religion		
Muslim	13.1%	13.2%
Hindu and others	86.9%	86.8%
Caste		
SC/ST†	28.9%	37.0%
OBC‡	44.1%	42.8%
Other caste/general	27.0%	20.2%
Place of residence		
Rural	66.4%	72.5%
Urban	33.6%	27.5%
Region of residence		
North	13.2%	9.1%
West	14.6%	7.9%
South	23.6%	25.5%
Northeast	3.4%	2.9%
East	22.9%	32.2%
Central	22.3%	22.3%

*Row per cents. All other per cents are column.

†Scheduled caste/scheduled tribe (legal designations of lower caste and indigenous groups).

‡Other backwards class (legal classification of socially and educationally disadvantaged groups).

### Iterative thematic analysis using lasso and ridge regression models

**Figure 2:**
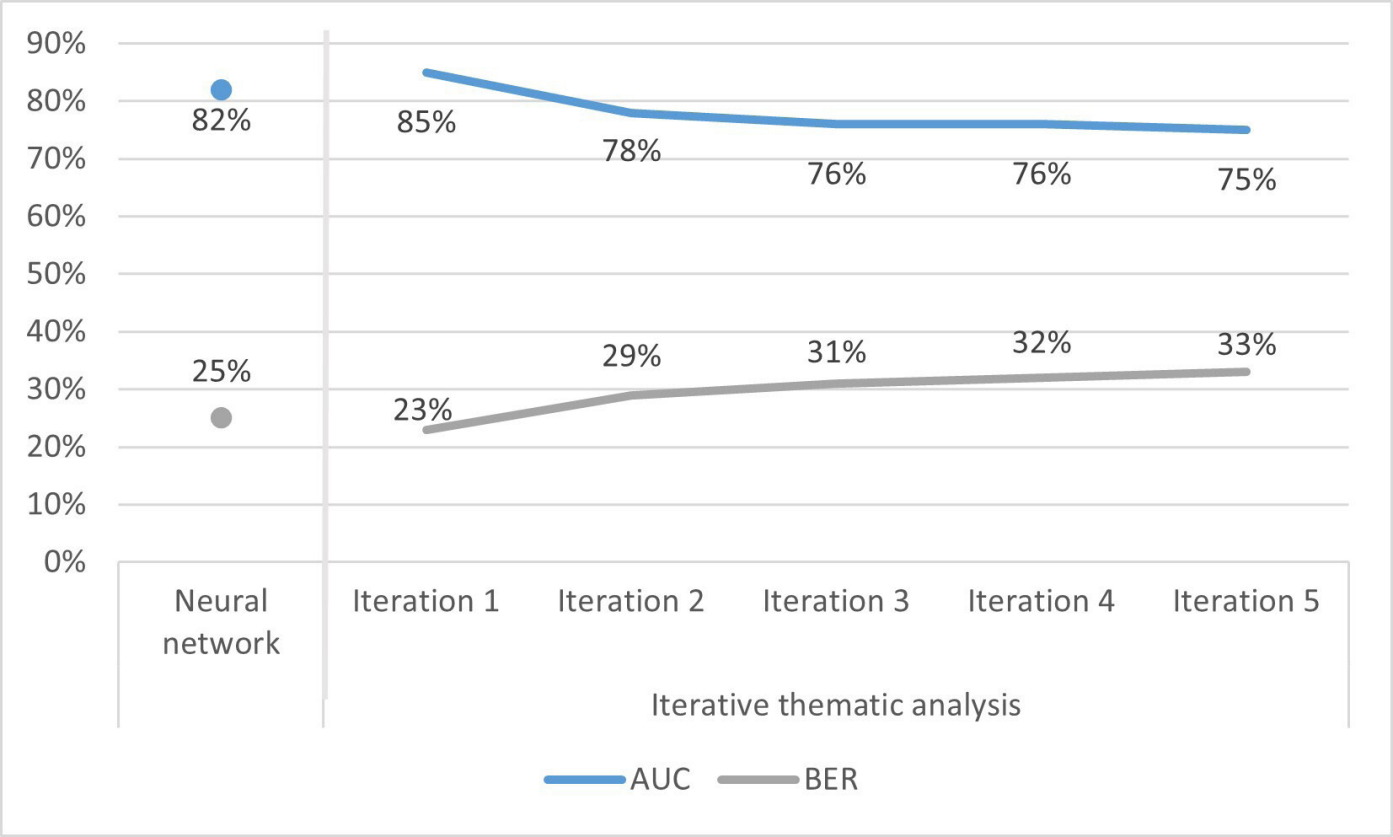
AUC and BER values for neural network and iterative thematic analysis models. BER, balanced error rate; AUC, area under receiver operating characteristic curve.

Models in the first iteration of analysis had had an AUC of 85%, and BER 23% (figure 2). The AUC varied from 75% to 78% in subsequent iterations; BER varied between 23% and 33%. Details on other evaluation metrices such as sensitivity, and specificity, are available in online supplemental table 1. The first iteration identified eleven themes: experiences of/exposure to violence (including marital and non-marital violence, abuse during pregnancy, intergenerational violence), sexual behaviour (including recent sexual activity, monogamy, early age of sexual initiation), decision making and freedom of movement (including lack of healthcare and financial agency, low freedom of movement, extended time away from home, ability to independently decide on family visits), sociodemographics (including low education and wealth levels for respondent and partner, marital duration of 10–14 years, scheduled caste, residence in select states (Bihar, Haryana, Jharkhand, Manipur), low-wage occupations), access to media (including regular consumption of television and radio), health knowledge (both low and high levels of health knowledge across different topics), health system interaction (permission and financial barriers hindering healthcare), partner control (partner has financial control, partner wants more children), economic agency (low levels of financial control, has taken microcredit loan), reproductive and maternal history (low levels of maternal and neonatal care, pregnancy complications, unwanted pregnancy), and health status (pregnancy complications, gynaecological concerns, terminated pregnancy, diabetes) (table 2). A full list of variables categorised into these and other identified themes is available in online supplemental table 2.

**Table 2:** Themes included/excluded within each round of iterative thematic analysis

Iteration 1	Iteration 2	Iteration 3	Iteration 4	Iteration 5
Excluded themes				
	Experiences of/exposure to violence	Experiences of/exposure to violence	Experiences of/exposure to violence	Experiences of/exposure to violence
		Substance abuse	Substance abuse	Substance abuse
			Sexual behaviour	Sexual behaviour
				Sociodemographics
Included themes				
Experiences of/exposure to violence	Substance abuse*	Sexual behaviour	Sociodemographics	Decision-making and freedom of movement
Sexual behaviour	Sexual behaviour	Sociodemographics	Decision-making and freedom of movement	Health system interaction
Decision-making and freedom of movement	Decision-making and freedom of movement	Decision-making and freedom of movement	Health system interaction	Health knowledge
Sociodemographics	Health system interaction	Health system interaction	Health knowledge	Diet
Access to media	Sociodemographics	Health knowledge	Diet	Economic agency
Health knowledge	Access to media	Diet	Access to media	Access to media
Health system interaction	Partner control	Economic agency	Partner control	Partner control
Partner control	Economic agency	Access to media	Economic agency	Fertility preferences
Economic agency	Diet*	Partner control	Fertility preferences	Reproductive and maternal history
Reproductive and maternal history	Health knowledge	Reproductive and maternal history	Reproductive and maternal history	Health status
Health status	Fertility preferences*	Health status	Health status	
	Reproductive and maternal history			
	Health status			

Themes included within iterations are illustratively ordered by the largest effect size of any variable within that theme in each iteration.

*New theme.

Variables in the experiences of/exposure to violence theme had the highest coefficient value in the first iteration, and were thus excluded in subsequent rounds. Three new themes were identified in the second iteration: substance abuse (partner consumes alcohol, smoking in respondent’s home), diet (never eats fruit, occasionally consumes dairy) and fertility preferences (husband wants more children, respondent wanted to delay pregnancy). The substance abuse theme contained the variable with the highest coefficient value, and was therefore excluded in subsequent rounds. The theme containing the variable with the highest coefficient value in the third iteration was sexual behaviour; for the fourth iteration, it was sociodemographics, and for the fifth iteration it was decision-making and freedom of movement. As no new themes were identified in the third, fourth or fifth iterations, the iterative thematic analysis process ended after the fifth iteration.

### Neural network models

The neural network model had AUC of 82% and BER 25% (figure 2). Variables identified through the neural network model are listed in box 1, and correspond to the following themes identified in iterative thematic analysis: decision-making and freedom of movement, diet, sociodemographics, health system interaction, health knowledge and experiences of/exposure to violence during childhood.

Box 1 **Variables in the neural network model above the coefficient curve knee point in the neural network model**

Getting medical help for self: getting permission to go is not a big problem.

Frequency eats fruits: occasionally.

Wealth index: middle.

Belongs to other backward class.

Getting medical help for self: not wanting to go alone is not a big problem.

Getting medical help for self: concern no drugs available is not a big problem.

HIV knowledge: a healthy-looking person can have HIV: yes.

Getting medical help for self: getting money needed for treatment is not a big problem.

Frequency eats dark green leafy vegetables: daily.

Husband/partner’s occupation (grouped): agricultural.

Syringe and needle from new, unopened package: yes.

Getting medical help for self: getting permission to go is not a big problem.

Respondent’s father ever beat her mother: no.

Someone smoked in respondent’s home or presence, in last 30 days: no.

Knows modern method of contraception: yes.

Knowledge on HIV: ways to avoid HIV/AIDS includes using condoms: no.

Knowledge on HIV: HIV transmitted by breastfeeding: yes.

Person who usually decides on respondent’s healthcare: respondent and husband/partner.

Person who usually decides on large household purchases: respondent and husband/partner.

Frequency of listening to radio: not at all.

Beating justified if wife neglects the children: no.

## Discussion

Nearly 6% of ever-married women aged 15–49 in India reported experiencing MSV over the past 12 months. Iterative thematic categorisation identified experiences of/exposure to violence as the variable most associated with MSV (as measured by the highest coefficient value) in the first model iteration. This is in line with a large volume of existing research on factors associated with marital violence in India indicating that marital violence is often perpetrated in multiple forms (eg, sexual, physical, emotional),^6 33–37^ and that violence is often intergenerational.^34 38 39^ This is particularly relevant for girls who marry early, as they are vulnerable to a sustained cycle of violence from childhood into their married adulthood.

In addition to experiences of/exposure to violence, several other themes emerged from iterative thematic analysis that reflect existing understanding of the factors associated with MSV in India and elsewhere. These include partner control (where variables indicate low levels of financial agency and husbands who want additional children),^40–42^ sociodemographics (where variables indicate highly marginalised populations, in terms of household poverty, scheduled caste membership, low levels of education for the woman and her partner, and work in informal, low-skill, low-wage occupations),^40–43^ and substance abuse (where variables indicate that the partner drinks alcohol).^21 42 44 45^ The fact that these known correlates emerged from this iterative thematic analysis process serves as an indicative mechanism that this approach is able to accurately identify important correlates, as is being increasingly demonstrated by evidence growing in this field.^26^

Importantly, the iterative thematic analysis process allowed new themes to emerge over the course of the five iterations of models that more traditional public health modelling approaches may not have considered. Women’s decision-making and freedom of movement proved important factors associated with MSV, in line with previous research identifying how women’s mobility patterns are associated with different forms of sexual violence.^12^ This theme, highlighting movement restrictions and compromised of medical and economic decision-making agency, in tandem with barriers to health system interactions, suggests that women with compromised agency in some family and community settings are also those who are experiencing MSV.

Health knowledge emerged as a complex theme. Women who experienced recent MSV tended to have lower levels of health knowledge (eg, believed TB was spread via sexual contact, never heard of oral rehydration, unaware of some sources of condoms), though this was paired with more accurate HIV-specific health knowledge. This may indicate that HIV-specific programmes have had more success in reaching populations affected by MSV than general health knowledge campaigns. Greater access to media through daily consumption of radio and TV messaging, was also associated with recent MSV, underscoring the suggestion that media campaigns may be successful in promoting violence prevention awareness and support services, though this should be paired and supported by direct outreach, screening and service provision.

Barriers to health, particularly sexual and reproductive health, were also associated with recent MSV. The themes of health system interaction (indicative of restricted agency and economic barriers), and reproductive and maternal history (including unwanted and terminated pregnancies, pregnancy complications and poor neonatal care) were significant in many rounds of iterative thematic analysis. India has recommended that frontline health workers be trained in recognising and addressing signs of domestic violence, but the efficacy of this approach is not well understood.^17 46^ Additionally, while some guidelines on the Indian health sector response to sexual violence exist, there are not comprehensive protocols for screenings to be included in all facility-based health interactions in India, and most medical staff do not receive the necessary training for widespread rollout of this approach.^17 18^ The results of this analysis suggest that health interactions can be an important point of identification for women experiencing MSV, and may be appropriate venues in which to provide resources and support for prevention and mitigation. While some components of this integration approach have been rolled out in smaller settings, their effectiveness, pathways and mechanisms merit careful evaluation prior to scale-up.^17 47^

Some of the emerging themes through iterative thematic analysis also need to be looked at in context of the nature of the behaviour itself. For example, the emergence of sexual behaviour as a theme is natural and expected, as in order have experienced recent martial sexual violence, women would have engaged in recent sexual activity. Variables within the sexual behaviour theme are indicative of monogamous relationships where the husband is the only lifetime sexual partner, and where age at first sex was early (15–17 years of age), in line with other research indicating linkages between child marriage and intimate partner violence in India.^19 48^

While the neural network model identified many factors significantly associated with recent MSV that were similar to those identified through iterative thematic analysis (particularly in the themes of sociodemographics, diet, decision-making and freedom of movement and experiences of/exposure to violence), it is difficult to summarise the results of these variables in terms of hypotheses suggestive of areas needing further research. It may be that this modelling approach is overly opaque for the current application, in contrast to iterative thematic analysis.

This study must be interpreted in light of important limitations. Data are cross-sectional, and the nature of the analysis does not allow for any inference around temporality or causality. Our results are specific to women aged 15–49 in India, and should not be assumed to apply elsewhere. Pathways and themes identified in this analysis need further unpacking in both qualitative and longitudinal research to better understand context, barriers, enablers and causal mechanisms. In addition, as with all individual response surveys, data are subject to recall and social desirability bias, especially given the sensitivity of the issue. Additionally, information on experiences of MSV are limited to those measures included in the NFHS-4 questionnaire, and thus omit some factors that would further inform our understanding of this outcome, including the place and specific circumstances of perpetration. While the machine learning modelling approaches used in this study have been previously validated, we again emphasise that the results from models such as these are designed to generate hypotheses rather than to pinpoint specific variables of importance.

MSV is a persistent violation of women’s bodily integrity and rights that has proven difficult to reduce at a population level. Exploring new factors that contribute to this abuse is an important pathway to complement and augment existing approaches to violence prevention, and the novel application of machine learning algorithms blended with qualitative thematic coding used in this analysis offers one such avenue. In addition to the known correlates of experiences of/exposure to other forms of violence and substance abuse, our results highlight the relationship of compromised agency in family and community settings, as well as health system interactions and adverse health status, with MSV. There may thus be opportunities to reach women during health encounters, and to target them with multi-pronged empowerment, health and violence support programming, supporting a more holistic approach to women’s well-being.

## Data Availability

Data are available in a public, open access repository. Data are publicly available at https://dhsprogram.com/data/dataset_admin/login_main.cfm.
