# Supplementary material for: Opening closed doors: using machine learning to explore factors associated with marital sexual violence in a cross-sectional study from India
Source: BMJ Open. 2021 Dec 30;11(12):e053603. doi: 10.1136/bmjopen-2021-053603 (PMC8718485; doi:10.1136/bmjopen-2021-053603)
Supplement: Supplementary data [file bmjopen-2021-053603supp001.pdf]

Supplementary Table 1. Sensitivity and specificity of iterative thematic analysis and neural network models

	Sensitivity	Specificity
Iterative Thematic Analysis		
Model 1	69.1994573	84.8868923
Model 2	63.7720488	76.7447457
Model 3	61.7367707	75.3008182
Model 4	61.8724559	74.8355527
Model 5	60.1085482	74.4184181
Neural network	70.963365	78.0683459

Supplementary Table 2. Themes that emerged in iterative thematic analysis and associated variables.

Theme	Variables
Experiences of/ exposure to violence	• Experienced any emotional violence<br>• Experienced physical violence perpetrated by husband/partner<br>• Respondent afraid of husband/partner most of the time<br>• Respondent ever physically hurt husband/partner when he was not hurting her<br>• Someone hurt respondent during pregnancy<br>• Respondent's father perpetrated IPV<br>• Someone other than husband physically hurt respondent
Sexual behavior	• Had sex with most recent partner at least a month ago<br>• Times in last 12 months had sex with most recent partner : more than 10 times<br>• Age at first sex : 15:17 years<br>• Reason for not having sex recently is not because tired or not in mood<br>• Relationship with most recent sex partner : spouse<br>• Total lifetime number of sex partners: one<br>• Number of sex partners, including spouse, in last 12 months: 1<br>• Time since last sex (in days): less than 30 days<br>• Number of sex partners, excluding spouse, in last 12 months: 0
Decision-making and freedom of movement	• Getting medical help for self: getting permission to go is big problem<br>• Person who usually decides what to do with money husband earns is husband/partner alone<br>• Not at all allowed to go to places outside this village<br>• Getting medical help for self: not wanting to go alone is not a big problem<br>• Lived away from home for more than one month in last 12 months<br>• Person who usually decides how to spend respondent's earnings is husband/partner alone<br>• Person who usually decides on visits to family or relatives is respondent alone
Socio-demographics	• State: Bihar<br>• Wealth index: Poorest<br>• Belongs to scheduled caste<br>• Relationship to household head: daughter<br>• State: Haryana<br>• Husband or Partner's education level: had no education<br>• State: Jharkhand<br>• Religion: Hindu<br>• Respondent's mother tongue: Manipuri<br>• Husband/Partner's occupation : skilled and unskilled manual

	• Wealth index - rural: poorer<br>• Cohabitation duration: 10-14 years<br>• State: Manipur<br>• Highest educational level: Primary<br>• Age: 30-34 years<br>• Respondent's occupation: Services/Household and domestic
Access to media	• Frequency of listening to radio : almost every day<br>• Frequency of watching television : almost every day<br>• Source of information about AIDS is not newspapers/magazines
Health knowledge	• Believes that tuberculosis is spread by: sexual contact<br>• Never heard of oral rehydration<br>• Does not know that public: govt. dispensary could be a source of condoms<br>• Has heard about STIs<br>• Has seen family planning message on a wall painting or hoarding<br>• Source of information about AIDS is not newspapers/magazines<br>• Source of information about AIDS is work place<br>• Believes that people can get HIV/AIDS from blood products or blood transfusions<br>• Believes that ways to avoid HIV/AIDS is to limit sex to one partner/stay faithful to one partner<br>• Knows that they can get a condom<br>• Have been told by a health provider about how to deal with side effects of contraception
Health system interaction	• Getting medical help for self: getting permission to go is big problem<br>• Getting medical help for self: getting money needed for treatment is big problem<br>• Did not go for antenatal care during most recent visit to health center<br>• Transport used by respondent to go to health facility for delivery : tempo/auto/tractor<br>• Getting medical help for self: not wanting to go alone is not a big problem<br>• Times met with an Auxiliary Nurse Midwife in last 3 months: none<br>• Number of months pregnant when registered: one<br>• Has sought treatment for diabetes<br>• Services/matters talked about in last 3 months with a health provider: not about supplementary food<br>• Has been told by a health provider about how to deal with side effects of contraception
Partner control	• Person who usually decides what to do with money husband earns is husband/partner alone<br>• Husband's desire for children: husband wants more children<br>• Person who usually decides how to spend respondent's earnings is husband/partner alone

Economic agency	• Has ever taken a loan, cash or in kind, from these programmes, to start or expand a business<br>• Person who usually decides what to do with money husband earns is husband/partner alone<br>• Getting medical help for self: getting money needed for treatment is a big problem<br>• Person who usually decides how to spend respondent's earnings is husband/partner alone
Reproductive and maternal history	• During pregnancy, had difficulty with daylight vision<br>• Had genital discharge in last 12 months<br>• Baby was not immediately wiped dry and wrapped without being bathed after birth<br>• Transport used by respondent to go to health facility for delivery: tempo/auto/tractor<br>• During pregnancy, had convulsions not from fever<br>• Number of months pregnant when registered: one<br>• Has had a terminated pregnancy<br>• Did not want pregnancy when became pregnant but wanted it later<br>• Marriage to first birth interval: more than 25 months
Health status	• During pregnancy, had difficulty with daylight vision<br>• Had genital discharge in last 12 months<br>• During pregnancy, had convulsions not from fever<br>• Has sought treatment for diabetes<br>• Has had a terminated pregnancy
Substance abuse	• Husband/partner drinks alcohol<br>• Someone smoked in respondent's home or presence, in last 30 days
Diet	• Never eats fruits<br>• Occasionally takes milk or curd
Fertility preferences	• Husband's desire for children: husband wants more children<br>• Did not want pregnancy when became pregnant but wanted it later

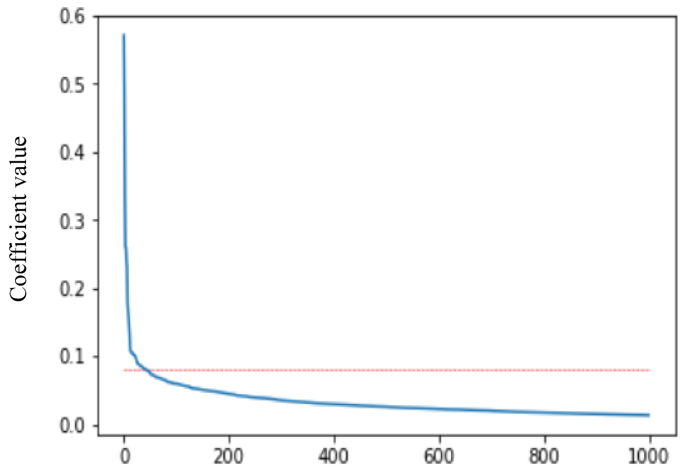

Supplementary Figure 1. Coefficient curve to identify knee-point (for Model 1 of iterative thematic analysis)
